# Disassembly of the JIP1/JNK molecular scaffold by caspase-3-mediated cleavage of JIP1 during apoptosis

**DOI:** 10.1016/j.yexcr.2011.01.011

**Published:** 2011-04-15

**Authors:** Mahesh Vaishnav, Marion MacFarlane, Martin Dickens

**Affiliations:** a Department of Biochemistry, Henry Wellcome Building, University of Leicester, Lancaster Road, Leicester LE1 9HN, UK; b Medical Research Council Toxicology Unit, Hodgkin Building, University of Leicester, P.O. Box 138, Lancaster Road, Leicester LE1 9HN, UK

**Keywords:** DEVD.fmk, benzyloxycarbonyl-Asp(OMe)-Glu(OMe)-Val-Asp(OMe) fluoromethyl ketone, DTT, dithiothreitol, EDTA, ethylene diamine tetra-acetic acid, FADD, Fas-associated death domain, GAP, GTPase activating protein, IB1, Islet Brain 1, JSAP, JNK/SAPK-associated protein, JIP, JNK interacting protein, JNK, c-jun amino-terminal kinase, MAPK, Mitogen-activated protein kinase, MAPKK, MAPK kinase, MAPKKK, MAPKK kinase, NF-κB, Nuclear factor κB, PARP, poly (ADP ribose) polymerase, PBS, phosphate buffered saline, RIP, Receptor interacting protein kinase, POSH, Plenty of SH3s, TNF, tumour necrosis factor, TRADD, TNF receptor associated death domain, TRAF, TNF receptor associated factor, TRAIL, TNF-related apoptosis inducing ligand, zVAD.fmk, benzyloxycarbonyl-Val-Ala-Asp(OMe) fluoromethyl ketone, JIP, JNK, Apoptosis, Caspase-3, TRAIL, HeLa

## Abstract

We report here the cleavage of the c-Jun N-terminal Kinase (JNK) pathway scaffold protein, JNK Interacting Protein-1 (JIP1), by caspases during both Tumour Necrosis Factor-Related Apoptosis-Inducing Ligand (TRAIL) and staurosporine-induced apoptosis in HeLa cells. During the initiation of apoptosis, maximal JNK activation is observed when JIP1 is intact, whereas cleavage of JIP1 correlates with JNK inactivation and progression of apoptosis. JIP1 is cleaved by caspase-3 at two sites, leading to disassembly of the JIP1/JNK complex. Inhibition of JIP1 cleavage by the caspase-3 inhibitor DEVD.fmk inhibits this disassembly, and is accompanied by sustained JNK activation. These data suggest that TRAIL and staurosporine induce JNK activation in a caspase-3-independent manner and that caspase-3-mediated JIP1 cleavage plays a role in JNK inactivation via scaffold disassembly during the execution phase of apoptosis. Caspase-mediated cleavage of JIP scaffold proteins may therefore represent an important mechanism for modulation of JNK signalling during apoptotic cell death.

## Introduction

Apoptosis is a conserved, intrinsic cellular suicide programme that is fundamental for the normal development and homeostasis of multicellular organisms [reviewed in [1,2]]. It is characterised by a distinct set of morphological and biochemical changes that ultimately result in the controlled dismantling and removal of the cell. The biochemical events have been divided into an initial commitment phase and an execution phase when the cell is physically dismantled and the characteristic morphological changes are observed. The commitment phase requires the proper integration of the survival and apoptotic signals that determine cell fate and in most contexts the execution phase depends upon the activity of caspases, a family of aspartate-specific cysteine proteases [reviewed in [3]]. Appropriate apoptosis therefore requires the close integration of cell signalling pathways with the core apoptotic machinery.

Mammalian caspases are divided into ‘initiator’ and ‘effector’ caspases. Initiator caspases (e.g. caspases-8 and -9) directly or indirectly activate effector caspases (e.g. caspases-3, -6 and -7). These effector caspases then cleave key intracellular substrates such as Poly (ADP-ribose) Polymerase (PARP) and lamins, resulting in the classical morphological features of apoptosis [3].

Caspases may be activated by both receptor (‘extrinsic’)- and non-receptor (‘intrinsic’)-mediated apoptotic pathways [reviewed in [2,4]]. The extrinsic pathway is activated by transmembrane proteins that belong to the tumour necrosis factor (TNF)/nerve growth factor (NGF) receptor superfamily. These include, the death receptors Fas (CD95/Apo1), TNFR-I and the two TNF-related apoptosis-inducing ligand (TRAIL) receptors, TRAIL-R1 and TRAIL-R2 [reviewed in [5]]. Fas, TNFR and TRAIL receptor ligation results in receptor trimerisation and binding of death domain-containing adaptor proteins such as FADD, TRADD, and TRAF2. FADD in turn recruits the apical caspase pro-caspase-8, thereby inducing formation of the Death-Inducing Signalling Complex (DISC) and autocatalytic activation of caspase-8.

The intrinsic apoptotic pathway is activated by a wide variety of toxic chemicals (e.g. staurosporine) and other stress-inducing stimuli [reviewed in [6]]. This pathway is characterised by the release of cytochrome *c* from mitochondria, which together with Apaf-1 and dATP results in formation of the ‘apoptosome’ and subsequent activation of pro-caspase-9. Caspase-9, in turn, activates effector caspases including caspases-3 and -7 and is therefore the primary initiator caspase for the intrinsic apoptotic pathway [reviewed in [4,7]].

The c-Jun N-terminal Kinase or JNK family of protein kinases are a group of enzymes that are activated by the exposure of cells to cytokines and a wide variety of environmental stresses. The proposed immediate upstream regulators of JNK are the MAP Kinase Kinases (MKKs) MKK4 and MKK7, which phosphorylate T183 and Y185 in the activation loop of JNK. Numerous MAP Kinase Kinase Kinases (MKKKs) that activate MKKs have been proposed [reviewed in [8–10]].

The role of JNK in apoptosis is complex and context-dependent. JNK activation is observed during apoptosis induced by a wide variety of stimuli in many cell types [10–14]. Studies using JNK deficient cells suggest that JNK activation is required for the proper regulation of stress-induced apoptosis. JNK activates the pro-apoptotic Bcl-2 family members Bax, Bid and Bim causing the release of cytochrome c and Smac/Diablo from mitochondria, leading to the activation of caspases via the “apoptosome” [10,15–22]. However, there is also good evidence to suggest that the JNK pathway may also contribute to cell survival in some circumstances [23–30].

A family of scaffold proteins, called JNK-Interacting Proteins (JIPs), has been identified [31–34]. These include JIP1 and JIP2 [also known as IB1/2 [35,36]] and JIP3/JSAP [33,37]. These proteins are thought to organise the components of the JNK pathway into specific signalling complexes [38]. Thus the JIP proteins are able to bind MLK3, MKK7 and JNK in a manner which may facilitate JNK signalling and activation [32,33,39,40].

The more recently identified scaffold protein JIP4, and the highly related JLP, appear to interact with both JNK and p38 [41,42]. Other scaffold proteins, such as POSH, may also be involved in the assembly of functional JNK/JIP-containing signalling complexes [43,44].

Mathematical modelling of MAPK pathway regulation by scaffold proteins shows that the level of MAPK activity is dependent on the level of expression of scaffold proteins in the cell [45,46]. Thus, the stoichiometry of the JIP signalling complexes involved in JNK activation depends on the level of JIP expression, and is likely to be important for determining JNK activity in the cell. Modulation of JIP levels may therefore provide a mechanism for the regulation of JNK activity and hence apoptosis in a context-dependent manner [40,47–52].

In a previous study we have shown that JNK activation during apoptosis induced by the proteasome inhibitor, lactacystin, is potentiated in the presence of caspase inhibitors [12]. In addition, inspection of the primary sequence of the JNK pathway scaffold protein JIP1, revealed putative caspase cleavage sites within the protein. These observations suggest that caspases may cleave the JNK pathway scaffold protein JIP1 to allow modulation of JNK activation during apoptosis.

We report here the cleavage of JIP1 by caspase-3 during receptor- and chemically-induced apoptosis in HeLa cells. Cleavage of JIP1 leads to disassembly of the JNK/JIP1 complex and correlates with JNK inactivation and progression of apoptosis. Caspase-mediated cleavage of JIP scaffold proteins may therefore represent an important mechanism enabling cells to switch off signalling by the JNK pathway once cells are committed to apoptosis.

## Materials and methods

### Cell culture and treatments

HeLa cells were maintained in Dulbecco's Modified Eagle Medium supplemented with 10% (v/v) foetal bovine serum, 2 mM L-Glutamine and 100 units/ml each of penicillin and streptomycin. Cells were grown in an atmosphere of 5% CO_2_ at 37 °C. Cells were plated onto 100 mm dishes, grown to 75% confluence and treated with TRAIL [53] or staurosporine (Sigma, UK), with or without 1 h pre-treatment with benzyloxycarbonyl-Val-Ala-Asp(OMe) fluoromethyl ketone (zVAD.fmk) (Enzyme Systems, Dublin, CA) or benzyloxycarbonyl-Asp(OMe)-Glu(OMe)-Val-Asp(OMe) fluoromethyl ketone (DEVD.fmk) (Alexis Biochemicals, Nottingham, UK) as described in the figure legends. Non-adherent cells were collected by centrifugation at 100 *g* at 4 °C for 3 min, washed once with ice-cold PBS and lysed in Triton Lysis Buffer (20 mM Hepes pH 7.5, 137 mM NaCl, 25 mM β-glycerol phosphate, 2 mM sodium pyrophosphate, 2 mM EDTA, 10% glycerol, 1% Triton X-100, 1 mM PMSF, 2.5 μg/ml each of pepstatin, antipain and leupeptin, and 2 mM benzamidine, 0.5 mM DTT, 1 mM Na_3_VO_4_). Attached cells were also washed once with ice-cold PBS and lysed in Triton Lysis Buffer. Lysates from non-adherent and adherent cells were pooled, clarified by centrifugation at 20, 000 *g* for 15 min at 4 °C, and the protein concentration determined by the method of Bradford [54].

### Quantification of apoptosis

For quantitative apoptosis measurements, cells were plated onto 22 × 22 mm glass coverslips and treated with the apoptotic agents for the times indicated in the figure legends. Non-adherent apoptotic cells were collected from the medium by centrifugation onto poly-L-lysine coated coverslips and processed for fluorescence microscopy alongside coverslips containing adherent cells. Cells were fixed with ice-cold methanol for 30 min at −20 °C and washed three times with calcium- and magnesium-free PBS before blocking in PBS/1% BSA at room temperature for 1 h. Cells were then washed five times with calcium- and magnesium-free PBS, and incubated with 1 μM Hoechst 33258 for 5 min at room temperature. Finally, cells were then washed three times with calcium- and magnesium-free PBS before mounting. The percentage of apoptotic cells was determined, using fluorescence microscopy, by counting the number of cells undergoing nuclear fragmentation as described previously [13].

### Western blot analysis

Cell lysates (200 μg) were subjected to SDS-PAGE and proteins transferred to Immobilon-P membranes (Millipore) by electrophoresis using a semi-dry transfer apparatus (Biorad). Western blots were probed with monoclonal antibodies to JIP1 (The kind gift of Dr. Roger Davis, HHMI, Worcester, USA), PARP (Pharmingen, cat. # 65196E), α-tubulin (Sigma, cat. # T5168), JNK1 (Pharmingen, cat. # 554286), and caspase-3 (kindly provided by Dr D. Nicholson, Merck Frosst), -7, -8 or -9 (kindly provided by Dr D. Green, Memphis) as indicated in the figure legends. Blots were further developed by incubation with appropriate HRP-conjugated secondary antibodies (Sigma) and visualised using enhanced chemiluminescence (GE Healthcare).

### In vitro translation and caspase cleavage reactions

[^35^S] Methionine-labelled JIP1 proteins were prepared by coupled in vitro transcription/translation of 2 μg of a pBluescript-JIP1 cDNA template, in a TNT® rabbit reticulocyte lysate, according to the manufacturer's instructions (Promega). For in vitro caspase cleavage reactions, a 10 μl aliquot of the transcription/translation mixture, containing [^35^S]-labelled JIP1, was incubated with purified recombinant caspase-3 or -8, as indicated, in a final volume of 25 μl containing 10 mM Hepes, pH 7.5, 2 mM EDTA, 0.1% CHAPS, 10 μg/ml antipain, 10 μg/ml pepstatin A, 20 μg/ml leupeptin, 2 mM PMSF, and 5 mM DTT [55]. The reactions were incubated at 30 °C for the times indicated and were terminated by the addition of SDS-PAGE sample buffer prior to SDS-PAGE on 10% gels. Gels were stained with Coomassie Brilliant Blue G250, destained, dried and the reaction products visualised by PhosphorImager analysis (GE Healthcare).

### JIP1 mutagenesis

Caspase-3 cleavage-resistant JIP1 mutants, DLIA and DESA and the double mutant DLIA/DESA were constructed by overlap extension PCR-mutagenesis with mismatch oligonucleotide primers using pBluescript-JIP1 as a template. All mutations were confirmed by automated DNA sequencing (Applied Biosystems).

### Immunoprecipitation and JNK Immune-complex kinase assays

After the indicated treatments and extraction, clarified HeLa cell lysates (400 μg) were pre-cleared with 5 mg of protein-A Sepharose (Sigma) for 1 h at 4 °C. Lysates were then incubated, for 3 h at 4 °C, with 5 mg Protein-A Sepharose and rabbit polyclonal antisera raised against full length JNK1. Immunoprecipitates were then washed three times with 1 ml of ice-cold Triton Lysis Buffer and either subjected to SDS-PAGE prior to western blotting for JIP1 or assayed for JNK activity. For JNK assays, the immunoprecipitates were further washed with 1 ml of Kinase Assay Buffer (25 mM Hepes pH 7.4, 25 mM β-glycerol phosphate, 25 mM MgCl_2_, 0.5 mM Na_3_VO_4_, and 0.5 mM DTT) and resuspended in Kinase Assay Buffer to a final volume of 50 μl containing 50 μM [γ -^32^P] ATP (2000 cpm/pmol) and 5 μg GST-c-Jun (1-79). After incubation for 30 min at 30 °C, assays were terminated by the addition of SDS-PAGE sample buffer, prior to electrophoresis on 10% SDS-PAGE gels. ^32^P-incorporation into GST-c-Jun was determined by PhosphorImager analysis of the dried gels (GE Healthcare).

## Results

### Caspase-mediated cleavage of JIP1 occurs during TRAIL and staurosporine-induced apoptosis of HeLa cells

The presence of putative caspase cleavage sites within the primary sequence of JIP1 raised the possibility that JIP1 might be cleaved during apoptosis to yield a protein product with potentially modified properties.

**Fig. 1:**
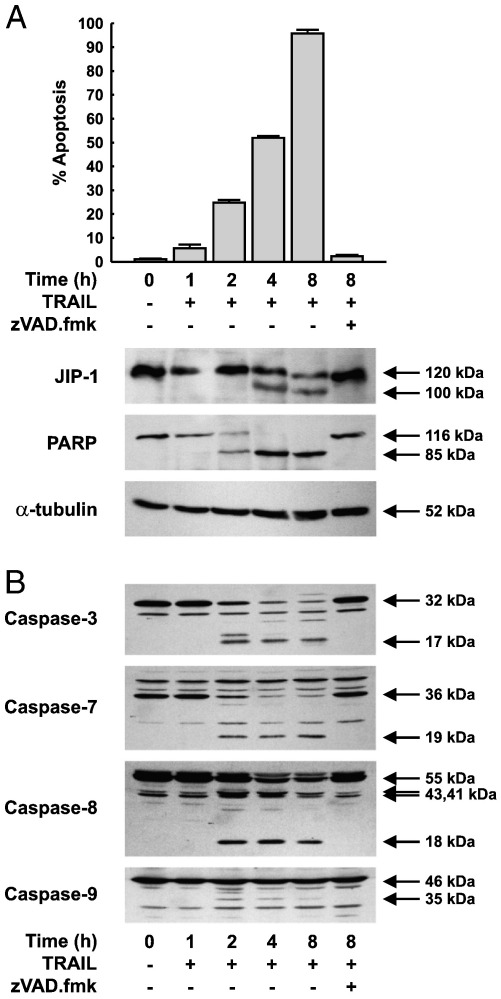
Caspase-mediated JIP1 cleavage during TRAIL induced apoptosis of HeLa cells. HeLa cells were treated with TRAIL (1 μg/ml) with or without 50 μM zVAD.fmk (added 1 h prior to the addition of TRAIL) for the times indicated. Adherent and non-adherent cells were collected, the percentage of apoptotic cells determined, and cell lysates immunoblotted with either monoclonal antibodies to JIP1, PARP and α-tubulin (A) or polyclonal antibodies raised against caspases-3, -7, -8 and -9 (B).

**Fig. 2:**
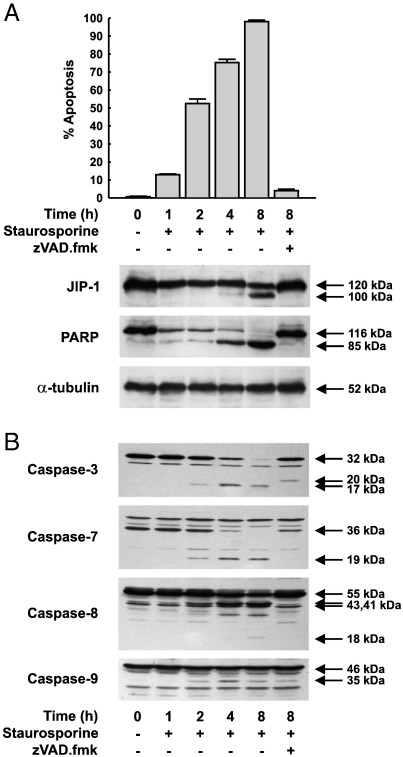
Caspase-mediated JIP1 cleavage during staurosporine induced apoptosis of HeLa Cells. HeLa cells were treated with staurosporine (1 μM) with or without 50 μM zVAD.fmk (added 1 h prior to the addition of staurosporine) for the times indicated. Adherent and non-adherent cells were collected, the percentage of apoptotic cells determined, and cell lysates immunoblotted with either monoclonal antibodies to JIP1, PARP and α-tubulin (A) or polyclonal antibodies raised against caspases-3, -7, -8 and -9 (B).

To test whether JIP1 is cleaved *in vivo* by caspases, HeLa cells were treated with either TRAIL (Fig. 1) or staurosporine (Fig. 2) in the presence or the absence of the general caspase inhibitor zVAD.fmk. The number of apoptotic cells was monitored and cell extracts subjected to western blotting for JIP1, PARP and α-tubulin (Figs. 1A and 2A).

Both TRAIL and staurosporine induced a time-dependent increase in the number of apoptotic cells in the culture that was inhibited by the general caspase inhibitor zVAD.fmk (Figs. 1A and 2A). In untreated HeLa cells, both JIP1 and PARP were present as intact polypeptides of 120 kDa and 116 kDa respectively. TRAIL caused the time-dependent loss of the 120 kDa, intact, form of JIP1 and the appearance of a 100 kDa fragment, first evident at 4 h, suggesting the proteolytic cleavage of JIP1 during apoptosis. Similar results were obtained in response to staurosporine treatment, although the kinetics were slower, with significant accumulation of the 100 kDa species evident only after 8 h. JIP1 cleavage in response to either stimulus was accompanied by a decrease in the amount of intact JIP1. However, in both cases, neither the complete loss of intact JIP1, nor the appearance of fragments smaller than 100 kDa was observed. Following exposure to both TRAIL and staurosporine, production of the 100 kDa fragment was completely blocked by the caspase inhibitor zVAD.fmk, indicating that cleavage of JIP1 *in vivo* during apoptosis is mediated by a caspase.

Treatment of HeLa cells with TRAIL or staurosporine caused a similar time-dependent cleavage of the caspase-3 substrate, PARP, which was cleaved to an 85 kDa fragment after 2 h in response to TRAIL (Fig. 1A), and after 1 h of staurosporine treatment (Fig. 2A). Complete loss of intact PARP was observed after 4 h of TRAIL and 8 h of staurosporine treatment. As expected, PARP cleavage was completely blocked by zVAD.fmk pre-treatment in response to either stimulus. Constant levels of α-tubulin throughout the time course, for both TRAIL and staurosporine treatment, confirmed equal protein loading and excluded the possibility that JIP1 cleavage might have been due to non-specific proteolysis.

The different kinetics of JIP1 and PARP cleavage in response to TRAIL and staurosporine suggested differential activation of caspases by these two stimuli. To test this, and to determine if the kinetics of JIP1 cleavage correlated with activation of a specific caspase, extracts of HeLa cells treated with TRAIL and staurosporine were examined for caspase activation by western blotting with antisera reactive to caspases -3, -7, -8 and -9 (Figs. 1B and 2B).

Caspases-3 and -7 were present primarily as their intact 32 kDa and 36 kDa proforms in control cells (Figs. 1B and 2B). Treatment with TRAIL or staurosporine resulted in the gradual loss of the inactive proforms and the concomitant appearance, first detected at 2 h, of their active 17 kDa and 19 kDa forms respectively (Fig. 1B). However, the kinetics of caspases-3 and -7 processing was slower in response to staurosporine, with a modest amount of their active fragments evident in cells treated with staurosporine compared with those treated with TRAIL (Fig. 2B). Staurosporine treatment for 4 h resulted in the efficient processing of caspases-3 and -7 and an increased formation of their active fragments that was comparable to that observed after 2 h of TRAIL treatment (Figs. 1B and 2B).

Caspase-8 was present primarily as two isoforms of very similar mobility, running at an apparent molecular weight of 55 kDa (Figs. 1B and 2B), corresponding to caspases-8a and -8b. Exposure to TRAIL or staurosporine resulted in the cleavage of caspase-8 initially to two fragments of 43 and 41 kDa. This was followed by the appearance of the active 18 kDa fragment, first observed 2 h after TRAIL treatment (Fig. 1B), but not evident until 8 h following exposure to staurosporine (Fig. 2B).

In untreated HeLa cells caspase-9 was evident as an inactive 46 kDa proform which, upon the induction of apoptosis, by either TRAIL or staurosporine, was processed in a time-dependent manner to yield the active 35 kDa fragment, which first appeared at 2 h (Figs. 1B and 2B).

Processing of all caspases to their corresponding active forms, in response to either stimulus, was significantly inhibited in cells pre-treated with the broad spectrum caspase inhibitor zVAD.fmk (Figs. 1B and 2B).

These results demonstrate that TRAIL and staurosporine show different kinetics of activation of both the initiator caspases (-8 and -9) and the effector caspases (-3 and -7). This was consistent with the differential kinetics of JIP1 cleavage observed in response to TRAIL and staurosporine in HeLa cells (Figs. 1A and 2A).

### JIP1 is a caspase-3 substrate in vivo

The potential caspase cleavage sites identified by inspection of the sequence of JIP1 most closely resembled the consensus sequence proposed for the major effector caspase, caspase-3 [3]. In addition, the kinetics of JIP1 cleavage in response to TRAIL and staurosporine are consistent with JIP1 being a caspase-3/-7 substrate.

**Fig. 3:**
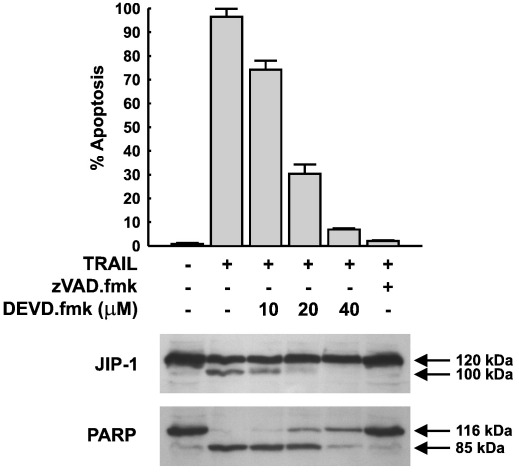
Effect of caspase inhibitors on JIP1 cleavage during TRAIL-induced apoptosis of HeLa cells. HeLa cells were treated with TRAIL (1 μg/ml) for 8 h in the presence or absence of either 50 μM zVAD.fmk or DEVD.fmk at the concentrations indicated. Caspase inhibitors were added 1 h prior to the addition of TRAIL. Adherent and non-adherent cells were collected and the percentage of apoptotic cells determined and lysates immunoblotted for JIP1 and PARP as indicated. Similar results were obtained with staurosporine (not shown).

To investigate the possibility that JIP1 might be a caspase-3/-7 substrate *in vivo*, HeLa cells were pre-treated with increasing concentrations of a more specific caspase-3/-7 inhibitor, DEVD.fmk, and exposed to TRAIL for 8 h. Extracts were then prepared and blotted for JIP1 and the caspase-3 substrate, PARP. In control cells JIP1 and PARP were present as 120 kDa and 116 kDa fragments respectively (Fig. 3). TRAIL treatment in the absence of DEVD.fmk resulted in the cleavage of JIP1 to a ~ 100 kDa product, and cleavage of PARP to an 85 kDa fragment. Increasing concentrations of DEVD.fmk resulted in the progressive inhibition of JIP1 cleavage, with complete inhibition seen at 40 μM (Fig. 3). In addition, we confirmed that 40 μM DEVD.fmk inhibited caspase-3 activity completely but had no effect on caspase-8 processing or activity (data not shown). Inhibition of JIP1 cleavage by DEVD.fmk was comparable to the inhibition achieved with zVAD.fmk. A concentration-dependent inhibition of PARP cleavage in the presence of DEVD.fmk was also observed, with 40 μM DEVD.fmk inhibiting PARP cleavage and thus demonstrating the complete inhibition of caspase-3 activity. Identical results were obtained for the inhibition of JIP1 cleavage by DEVD.fmk in staurosporine treated HeLa cells (not shown). These results clearly demonstrate that JIP1 is a caspase-3 substrate *in vivo*.

### Preferential cleavage of JIP1 by caspase-3 in vitro

**Fig. 4:**
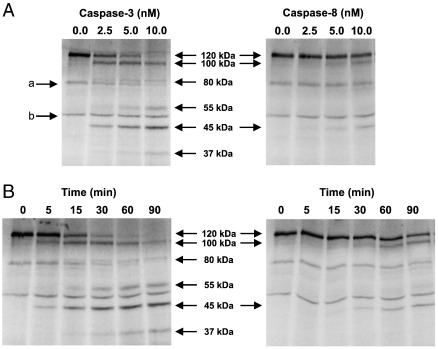
Cleavage of JIP1 by caspases-3 and -8 in vitro. ^35^S-labelled, in vitro translated JIP1 was incubated with either (A) varying concentrations of purified caspase-3 or -8 for 1 h at 30 °C or (B) a fixed concentration of caspase-3 or -8 (10 nM) for the times indicated. The reaction products were visualised by SDS-PAGE and autoradiography. The migration of two minor translation products (a and b) are indicated. Band a is likely to have arisen due to the use of an alternative internal translational initiation site. The identity of band b is unknown, but appears to be unrelated to JIP1 since its intensity is unaffected by caspase treatment.

To confirm that JIP1 was a caspase-3 substrate, and to detect additional JIP1 cleavage products not detected by our JIP1 antibody, we incubated ^35^S-labelled JIP1, prepared by in vitro transcription/translation, with increasing concentrations of purified recombinant caspase-3 in vitro (Fig. 4A). Purified recombinant caspase-8 was used for comparison.

In the absence of purified recombinant caspases, JIP1 was present as an intact 120 kDa polypeptide. Minor bands, migrating at ~ 80 kDa and ~ 50 kDa, marked as **a** and **b** respectively in Fig. 4A, were also present. Upon incubation with 2.5 nM caspase-3, in addition to the intact 120 kDa JIP1 polypeptide, fragments of ~ 100, ~ 55 and ~ 45 kDa were also detected (Fig. 4A). At very high concentrations of caspase-3 a minor 37 kDa species was also detected. The 100 kDa JIP1 fragment detected in vitro is very likely to correspond to the 100 kDa JIP1 species observed during apoptosis *in vivo,* which is detected by immunoblotting. Increasing concentrations of caspase-3 resulted in the loss of the 120 kDa and 100 kDa fragments and an increase in the formation of the 55 kDa and 45 kDa products. The 100 kDa and 45 kDa products were not detected upon incubation with purified recombinant caspase-8 at the equivalent concentration of 2.5 nM. Products of this size were only just detectable even upon incubation with caspase-8 at higher concentrations. An essentially identical set of fragments were obtained in a time course of digestion at a fixed caspase concentration of 10 nM (Fig. 4B). These results demonstrate that JIP1 is an efficient caspase-3 substrate in vitro and is cleaved preferentially by caspase-3 rather than caspase-8.

### Cleavage of JIP1 by caspase-3 occurs in vitro at two sites

**Fig. 5:**
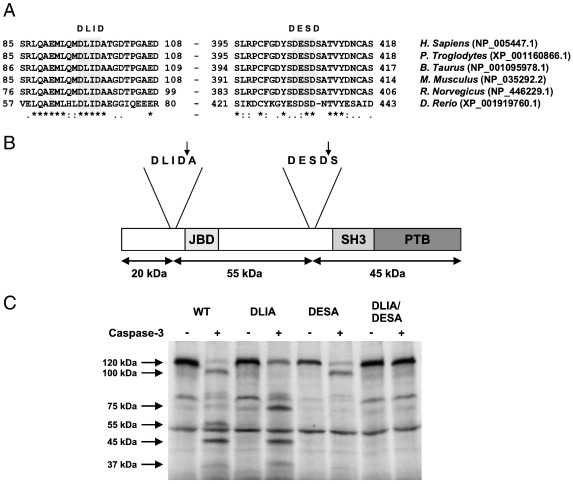
Identification of caspase-3 cleavage sites in JIP1. (A) Sequence alignment showing conserved caspase-3 cleavage sites in JIP1 from various species. (B) A diagram of JIP1 indicating the proposed sites of caspase-3 cleavage and the sizes of the expected cleavage products. Also shown are the positions of the JNK binding domain (JBD), Src homology 3 domain (SH3) and phosphotyrosine binding domain (PTB). (B) Wild-type and cleavage-resistant JIP1 mutants DLIA, DESA and the double mutant DLIA/DESA were ^35^S-labelled by in vitro translation and incubated with or without 10 nM caspase-3 for 1 h at 30 °C. The reaction products were visualised by SDS-PAGE and autoradiography.

The formation of three major products (100 kDa, 55 kDa and 45 kDa) on incubation of JIP1 with caspase-3 suggests two major cleavage sites. Inspection of the amino acid sequences for JIP1 indicate two conserved putative caspase-3 cleavage sites with sequences DLID and DESD (Fig. 5A). The size of the fragments produced by caspase-3 digestion is entirely consistent with cleavage occurring preferentially at these sites (Fig. 5B). To confirm this directly we replaced the aspartic acid residues at the cleavage site with alanine by site-directed mutagenesis (DLI*D* → DLI*A*, DES*D* → DES*A* and the double mutant DLI*A*/DES*A*) and tested their efficiency as substrates for caspase-3 in vitro (Fig. 5C).

Equivalent amounts of ^35^S-labelled, in vitro translated JIP1 (wild-type), JIP1 (DLIA), JIP1 (DESA) and JIP1 (DLIA/DESA) were incubated in the presence or the absence of 10 nM purified caspase-3, and the reaction products visualised by SDS-PAGE and autoradiography. In the absence of purified caspase-3, the wild-type and mutant JIP1 proteins were present as full length 120 kDa polypeptides (Fig. 5B). Minor translation products were also detected running at approximately 80 kDa and 50 kDa as seen previously (Figs. 4A and B). As expected, wild-type JIP1 was cleaved by caspase-3 to yield three major products of approximately ~ 100 kDa, ~ 55 kDa and ~ 45 kDa, with a considerable loss of the intact protein (Fig. 5B). Incubation of the in vitro translated JIP1(DLIA) with purified caspase-3 did not result in the formation of the 100 kDa and 55 kDa products produced from the wild-type JIP1. However, the 45 kDa product was still evident and there was a significant increase in the production of a 75 kDa species. Cleavage of the in vitro translated JIP1(DESA) with purified caspase-3 led to a significant loss of the intact protein and the formation of the 100 kDa product, but the 45 kDa and 50 kDa products were not detected (Fig. 5B). Neither was the 75 kDa product detected on cleavage of the DESA mutant by caspase-3. The JIP1 double mutant (DLIA/DESA) was entirely resistant to cleavage by caspase-3. Taken together these data confirm that JIP1 is a caspase-3 substrate in vitro and that the preferred cleavage sites are DLID and DESD, with no additional sites.

### The caspase-3 inhibitor DEVD.fmk potentiates JNK activation during TRAIL and staurosporine induced apoptosis

**Fig. 6:**
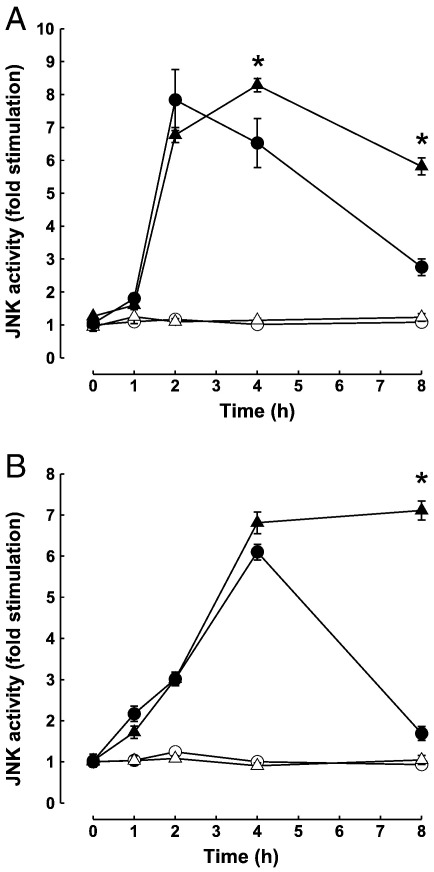
Effect of caspase-3 inhibition on JNK activation by TRAIL and staurosporine in HeLa cells. HeLa cells were treated with (closed symbols; ●, ▲) or without (open symbols; ○, △) 1 μg/ml TRAIL (A) or 1 μM staurosporine (B) in the presence (triangles; △, ▲) or absence (circles; ○, ●) of 40 μM DEVD.fmk. At the time points indicated, cell lysates were prepared from adherent and non-adherent cells, and JNK activity measured by immune-complex kinase assay as described in Experimental Procedures. The results shown represent the mean ± SEM for four independent experiments. Data points marked by asterisks (*) indicate statistically significant differences at p < 0.05 in a two-tailed *t*-test.

JNK activation precedes the induction of, and contributes to, apoptosis in many cell types. Since JIP1 has been implicated as a scaffold protein that organises and regulates the activity of the JNK module [39] its cleavage by caspase-3 may have consequences for the activity of the JNK pathway during apoptosis. To test the hypothesis that JIP1 cleavage during apoptosis may lead to changes in JNK activity we measured the JNK activity profile during TRAIL and staurosporine-induced apoptosis of HeLa cells in the presence and absence of the caspase-3 inhibitor, DEVD.fmk (Figs. 6A and B).

HeLa cells were treated with TRAIL or staurosporine, in the presence or absence of 40 μM DEVD.fmk, and cell extracts prepared from adherent and non-adherent cells were assayed for JNK activity (Fig. 6). Throughout the time course JNK activity remained at basal level in control cells and also in cells treated with DEVD.fmk alone (Figs. 6A and B). Consistent with our previous observations [12] TRAIL caused an ~ 8-fold increase in JNK activity at 2 h, followed by a steady decline to ~ 3-fold at 8 h (Fig. 6A). In staurosporine-treated cells, JNK activity increased more slowly, reaching a maximum of 6-fold above the basal level after 4 h (Fig. 6B). However, on reaching this peak it declined rapidly such that the JNK activity observed after 8 h was only ~ 2-fold above the basal level (Fig. 6B). In both TRAIL and staurosporine treated cells the decline in JNK activity after the initial peak, at either 2 or 4 h respectively, was markedly attenuated in the presence of the caspase-3 inhibitor DEVD.fmk. In cells incubated with TRAIL and DEVD.fmk, JNK activity was significantly elevated at 4 h and 8 h when compared to cells treated with TRAIL alone (p < 0.05; Fig. 6A). Although in staurosporine treated cells, no effect of DEVD.fmk was evident at 4 h, at 8 h JNK activity in the presence of the inhibitor was significantly higher than in cells treated without DEVD.fmk (p < 0.05; Fig. 6B). This led to the maintenance of JNK activity at levels that were even higher than the maximal activity seen in cells treated with staurosporine alone (Fig. 6B).

These results demonstrate a correlative relationship between JNK activation and JIP cleavage. In both TRAIL and staurosporine treated HeLa cells, JNK activation reached its maximum at 2 h and 4 h respectively, immediately before the first detectable cleavage of JIP1 by caspase-3 (4 h, Figs. 1, 2 and 6). The subsequent decrease in JNK activity closely matched the kinetics of JIP1 cleavage and both JIP1 cleavage and the decline in JNK activity were blocked by pre-treatment with the caspase-3 inhibitor DEVD.fmk.

### Cleavage of the JIP1 protein by caspase-3 causes disassembly of the JNK/JIP1 complex

**Fig. 7:**
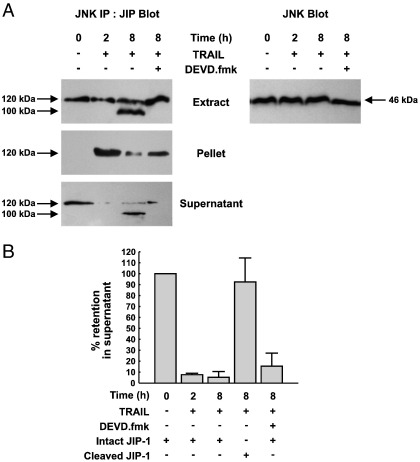
Co-immunoprecipitation of JIP1 with JNK during apoptosis in HeLa cells. (A) HeLa cells were treated with or without 1 μg/ml TRAIL in the presence or absence of 40 μM DEVD.fmk. At the time points indicated, cell lysates were prepared from adherent and non-adherent cells. Extracts were either immunoprecipitated with anti-JNK antibody and extract, immunoprecipitate and supernatant western blotted for JIP1, (left panel) or the extract alone blotted for JNK (right panel). (B) Western blots showing JNK and JIP1 co-immunoprecipitation were quantified by densitometry and the data expressed as percentage retention of JIP1 in the supernatant.

The JIP proteins have been proposed to facilitate the activation of the JNK pathway by forming signalling complexes containing the upstream kinases required for JNK activation. The decrease in JNK activity observed at the onset of JIP1 cleavage by caspase-3 suggests that JIP1 is required for full activation of the JNK pathway in response to TRAIL and staurosporine. To test if cleavage of JIP1 during apoptosis results in disassembly of the JIP1/JNK complex we examined the co-immunoprecipitation of endogenous JIP1 with JNK in HeLa cells exposed to TRAIL in the presence or absence of DEVD.fmk. Western blotting of control cell extracts shows that both JIP1 and JNK are readily detected (Fig. 7A, top panel). As expected, the addition of TRAIL resulted in the time-dependent cleavage of JIP1 to a 100 kDa fragment, detectable in the cell extract at 8 h, which was blocked by DEVD.fmk (Fig. 7A, top panel). Neither TRAIL nor DEVD.fmk incubation had any effect on the level of JNK detected in the cell extract during the time-course of the experiment (Fig. 7A, top right panel). In control cells, no JIP1 was detected in JNK immunoprecipitates (Fig. 7A, middle panel), with JIP1 remaining in the supernatant (Fig. 7A, lower panel), thus indicating no interaction between JNK and JIP1 in un-stimulated cells. After stimulation with TRAIL for 2 h, JIP1 and JNK efficiently co-precipitated, leaving little JIP1 remaining in the supernatant. At this time point no cleavage of JIP1 was observed (Figs. 1A and 7A). However, after 8 h of TRAIL treatment, by which time significant JIP1 cleavage was observed, the co-precipitation of JIP1 and JNK was markedly decreased compared to that seen at 2 h, with significant immunoreactivity remaining in the supernatant (Fig. 7). In addition, the JIP1 that did co-precipitate with JNK at this time point was the intact 120 kDa form of JIP1, and not the cleaved 100 kDa form which was instead left behind in the supernatant. In the presence of the caspase-3 inhibitor, DEVD.fmk, the cleavage of JIP1 in response to TRAIL was blocked and JIP1 and JNK co-precipitation was restored (Fig. 7). Taken together these observations show that the interaction of JNK and JIP1 is dependent on the presence of the intact 120 kDa form of JIP1 and that caspase-3-mediated JIP1 cleavage results in disassembly of the JIP1/JNK complex.

## Discussion

We have previously shown enhanced JNK activation in response to caspase inhibition during chemically-induced apoptosis [12]. An inspection of the JIP1 protein sequence shows several potential caspase cleavage sites [3]. This led us to believe that JNK activity in apoptotic cells may be modulated by caspases due to proteolytic cleavage of the JNK pathway scaffold protein JIP1. We investigated this possibility in HeLa cells treated with TRAIL or staurosporine, where we showed JIP1 to be cleaved to a 100 kDa fragment during apoptosis by either stimulus. JIP1 cleavage correlated with the progression of apoptosis, cleavage of the caspase-3 substrate, PARP and activation of caspases. Its appearance was blocked by the general caspase inhibitor zVAD.fmk and more specifically by the caspase-3 inhibitor DEVD.fmk.

These data clearly demonstrate that JIP1 is a target for caspases during apoptosis, and that cleavage of JIP1 *in vivo* is likely to be mediated by caspase-3. To confirm the specificity of caspase cleavage, and to detect additional JIP1 fragments generated by caspase cleavage, but not observed by western blotting, we tested the ability of caspase-3 and caspase-8 to cleave in vitro translated JIP1 protein. These experiments showed that JIP1 was preferentially cleaved by caspase-3 and was a poor substrate for caspase-8. Caspase-3 digestion resulted in the production of three JIP1 fragments of sizes consistent with cleavage at two sites (DLID and DESD). Cleavage at these sites was confirmed in vitro by site directed mutagenesis and we found no evidence for cleavage at other sites.

The JNK pathway is activated during apoptosis in many cell types and this may be facilitated by the JIP family scaffold proteins. If JIP1 were cleaved by caspases during apoptosis we would expect this to affect JNK activation. To determine if JIP1 cleavage during apoptosis had a functional consequence we measured the JNK activity in cells undergoing apoptosis in response to TRAIL or staurosporine in the presence and absence of the caspase-3 inhibitor DEVD.fmk. These experiments showed, as expected, activation of JNK by TRAIL and staurosporine. This activation was, however, transient, occurring during the initiation phase of apoptosis, with JNK activity declining after approximately 2–4 h and returning almost to basal levels at 8 h. The decline in JNK activity in both TRAIL and staurosporine treated cells correlated well with the cleavage of JIP1 by caspases. Furthermore, the inactivation of JNK later in the time-course was blocked in the presence of DEVD.fmk, leading to a sustained JNK activation in response to both TRAIL and staurosporine. This suggests that caspase-mediated cleavage of JIP1 may contribute to a decrease in JNK activity during the execution phase of apoptosis.

JIP scaffold proteins are believed to potentiate JNK activation by forming multi-protein signalling complexes containing JNK and its upstream activators [32,33,39,40]. However, the level of JIP expression seems to be critically important in determining the stoichiometry of such signalling complexes and either increased or decreased expression of JIPs can inhibit JNK signalling [45,46]. Indeed, our attempts to test the effect of caspase-resistant JIP1 mutants on JNK activation and apoptosis in HeLa cells were thwarted since exogenous expression of either wild-type or caspase-resistant JIP1 was sufficient to inhibit JNK signalling and block apoptosis (data not shown).

There are also good experimental correlations between JIP levels and JNK activity in many cell types [31,40,47–52]. Disassembly of JIP/JNK signalling complexes due to caspase cleavage of JIP1 could therefore contribute to the JNK inactivation we observe during the execution phase of apoptosis. We tested this by co-immunoprecipitation of endogenous JIP1 and JNK in cells undergoing apoptosis. The interaction of JNK with JIP1 was not observed in unstimulated cells, but was readily observed at early time points during apoptosis, before JIP1 cleavage was detected. At later time points, when there was significant JIP1 cleavage, JNK did not to co-precipitate with the cleaved 100 kDa fragment of JIP1, which was left behind in the supernatant. However, the intact JIP1 remaining at this time point was still able to bind JNK. Co-immunoprecipitation was restored in the presence of the caspase-3 inhibitor DEVD.fmk. This clearly shows that cleavage of JIP1 by caspases results in disassembly of the JIP1/JNK signalling complex even though the JNK binding domain, which is C-terminal to the caspase cleavage site at DLID, is still intact. Cleavage at the DESD site will separate the JNK binding domain from the binding site for the MLK family of kinases, situated towards the C-terminus of JIP1 in the PTB domain, and potentially that for MKK7, thus uncoupling JNK from its upstream activators [39,56]. Binding sites for other molecules that may also be involved in JNK regulation or affect its cellular location, such as kinesin, MKP-7 and POSH may also be lost [44,57,58]. Consistent with the observations of others [59], we find that JNK is not a direct target of caspases in this system (Fig. 7A) and JNK inactivation at late time points is therefore not a consequence of JNK proteolysis.

The fact that we do not see cleavage of all of the cellular JIP1 to the 100 kDa fragment during apoptosis suggests that there may be separate, caspase-sensitive and insensitive pools of JIP1. Considering the dynamic nature of JIP scaffold complexes that is now emerging, where the activation state of the pathway may determine which kinases are bound [see Fig. 7 and [60]], and the abundance of proposed binding partners, including MKK7, MLK3 and JNK, it is possible that the accessibility of JIP1 to caspases may be determined by the composition and activity of components of the complex with some binding partners protecting JIP1 from caspases. This may explain why we are able to detect additional JIP1 cleavage products by digestion of JIP1 with caspases in vitro*,* which are not apparent *in vivo*.

The cleavage of JNK pathway scaffold proteins by caspases is not restricted to JIP1. JIP3, also known as JSAP1, is cleaved by caspase-3 during apoptosis in NGF-deprived PC12 cells [33]. Inspection of the amino acid sequences of JIP2 and the more recently identified JIP4/JLP scaffold also reveal potential caspase cleavage sites. However, neither JIP2 nor JIP4 have yet been confirmed as caspase substrates experimentally and further work will be required to clarify this issue. Other MAPK scaffold proteins also appear to be targets for caspases. The ERK pathway scaffold, KSR1 is cleaved at a single site by caspase-3 during apoptosis [61], resulting in a loss of scaffold activity by separation of the MEK and ERK binding sites. This leads to down-regulation of ERK-mediated survival signalling and thus promotion of apoptosis [61]. These observations suggest that caspase-mediated cleavage of scaffold proteins is likely to be a general mechanism for the modulation of MAPK signalling during apoptosis.

Caspase-mediated cleavage of some signalling proteins, including MEKK1 [62,63], Ras GAP [64] and PKCδ [65], can directly promote apoptosis due to the production of pro-apoptotic proteolytic fragments. More commonly, caspases may promote apoptosis by inactivating proteins involved in signalling for cell survival. The cleavage of KSR1, mentioned above, is a good example. Others include the adapter proteins RIP and TRAF1 which when cleaved produce fragments which inhibit NF-kB activation, promoting apoptosis [66–69]. Similarly, caspase-mediated cleavage of Raf [59], Akt/PKB [70] and FAK [71,72] also promote apoptosis by inactivating cell survival pathways.

Since JNK activation during apoptosis is generally thought to contribute to cell death, where it appears to be required for stress-induced activation of caspases, cleavage of a JNK scaffold protein by caspases at first appears puzzling. However, the situation is likely to be more complex. There are numerous reports that JNK signalling in some instances may provide survival signals to counteract cell death [23–30], perhaps acting via JunD/NF-kB-mediated expression of the inhibitor of apoptosis cIAP-2 [26]. The role of JNK in regulating apoptosis is also likely to depend on the cell type, as well as the context and timing of JNK activation. In the initiation phase of apoptosis, the extent of JNK activation, and hence sensitivity of cells to apoptotic stimuli, may be dependent on JIP levels. JNK activation appears to be required to enter the execution phase, probably via activation of the mitochondrial pathway. However once caspases have been activated and the cell is committed to apoptosis, JNK activity may no longer be required. Our data suggest that caspase-mediated cleavage of JIP1 may contribute to the attenuation of JNK activity at later time points. This might prevent JNK exerting unwanted protective/survival effects late during apoptosis, thus committing the cell to death.

## Conclusion

In summary, our data show JIP1 to be a target for caspase-3 mediated cleavage in response to both chemical and receptor-mediated apoptotic stimuli. Caspase-3 cleaves JIP1 at two sites leading to the disassembly of the JNK/JIP signalling complex thought to be required for JNK activation. Caspase-3-mediated cleavage of JIP1 may play a role in attenuating JNK activity late during the execution phase of apoptosis, when its activity may no longer be required. Cleavage of both ERK and JNK scaffold proteins by caspases suggests a general mechanism for the regulation MAPK signalling during apoptotic cell death.
